# Complement activation in multiple sclerosis plaques: an immunohistochemical analysis

**DOI:** 10.1186/2051-5960-2-53

**Published:** 2014-05-09

**Authors:** Gillian Ingram, Sam Loveless, Owain W Howell, Svetlana Hakobyan, Bethan Dancey, Claire L Harris, Neil P Robertson, James W Neal, B Paul Morgan

**Affiliations:** Institute of Psychological Medicine and Clinical Neuroscience, Cardiff, UK; Institute of Infection and Immunity, School of Medicine, Cardiff University, Cardiff, UK; Department of Neurology and Molecular Neuroscience, Institute of Life Sciences, Swansea University, Swansea, UK

**Keywords:** Multiple sclerosis, Demyelination, Complement, Immunology, Histology, Immunohistochemistry

## Abstract

**Introduction:**

Inflammation and complement activation are firmly implicated in the pathology of multiple sclerosis; however, the extent and nature of their involvement in specific pathological processes such as axonal damage, myelin loss and disease progression remains uncertain. This study aims to bring clarity to these questions.

**Results:**

We describe a detailed immunohistochemical study to localise a strategically selected set of complement proteins, activation products and regulators in brain and spinal cord tissue of 17 patients with progressive multiple sclerosis and 16 control donors, including 9 with central nervous system disease. Active, chronic active and chronic inactive multiple sclerosis plaques (35 in total) and non-plaque areas were examined.

Multiple sclerosis plaques were consistently positive for complement proteins (C3, factor B, C1q), activation products (C3b, iC3b, C4d, terminal complement complex) and regulators (factor H, C1-inhibitor, clusterin), suggesting continuing local complement synthesis, activation and regulation despite the absence of other evidence of ongoing inflammation. Complement staining was most apparent in plaque and peri-plaque but also present in normal appearing white matter and cortical areas to a greater extent than in control tissue. C1q staining was present in all plaques suggesting a dominant role for the classical pathway. Cellular staining for complement components was largely restricted to reactive astrocytes, often adjacent to clusters of microglia in close apposition to complement opsonised myelin and damaged axons.

**Conclusions:**

The findings demonstrate the ubiquity of complement involvement in multiple sclerosis, suggest a pathogenic role for complement contributing to cell, axon and myelin damage and make the case for targeting complement for multiple sclerosis monitoring and therapy.

**Electronic supplementary material:**

The online version of this article (doi:10.1186/2051-5960-2-53) contains supplementary material, which is available to authorized users.

## Introduction

Multiple sclerosis (MS) is a chronic neuroinflammatory disease characterised by well demarcated white matter (WM) plaques of demyelination with varying degrees of axonal loss [1]. It is widely accepted that the disease process is initiated by adaptive immunity through infiltration of activated T cells into the CNS with associated up-regulation of proinflammatory mediators. However, the lack of lymphocyte recruitment after the initial relapsing phase of disease indicates that other factors drive pathology in late stage disease and there is increasing evidence implicating plasma cells and innate immunity in progressive disease [2].

Complement plays a central role in the innate immune system, providing an important defence against infection and immune complex disease. The system consists of approximately 30 circulating and membrane expressed proteins and is predominantly activated via the classical, alternative pathway or lectin pathways. These converge on a common effector pathway with formation of membrane attack complex (MAC) (which disrupts and forms pores in the phospholipid bilayer of a target cell), opsonins (molecules that enhance the ability of macrophages and neutrophils with complement receptors to phagocytose material - C3b, iC3b, C4b etc.) and anaphylatoxins (peptides that induce local and systemic inflammatory responses, increasing the permeability of blood vessels and attracting neutrophils through their chemotactic properties - C3a, C4a and C5a). Inappropriate activation of complement is normally prevented by complement inhibitors. A role for the complement system in MS has been established and is reviewed in Ingram 2010 [3]. Evidence implicating complement in MS came from studies measuring complement proteins in MS CNS tissue, plasma and CSF [4–6]. Numerous immunohistochemistry studies described the presence of complement proteins in and around areas of pathology in MS WM; however, analyses were generally limited to one or a few complement proteins and small numbers of cases [7–13]. Together, the published studies do not build consensus on whether complement protein immunoreactivity is a consistent feature in MS, which areas of the CNS demonstrate immunoreactivity, or which complement proteins and pathways are involved. This is likely due to studies including tissue from different stages of the disease, limited numbers of patients examined and variability in quality of detection reagents used. The cumulative evidence suggests that only tissues from cases with established and progressive disease demonstrate consistent complement staining [11]. In addition very few studies have attempted to define underpinning mechanisms of complement involvement in MS and its contribution to the various components of demyelination and axonal damage. A notable exception was a study by Prineas who showed abundant C3d deposition on myelin in areas of pathology and suggested a direct role as an opsonin driving immune response to myelin antigens [9].

The paucity of robust data and the lack of consensus noted above provoked us to look again at complement proteins in MS CNS tissue. We describe a systematic immunohistochemical analysis of the presence and localisation of key complement components, activation products, regulators and receptors in tissue from well-documented progressive MS. We find that the presence of complement proteins and activation products in and around areas of pathology is a consistent feature of progressive MS, although the extent varies between individuals. We note in particular, universal C1q staining indicative of classical pathway activation, and consistent staining for complement activation products, including in most cases the terminal complement complex (TCC), indicating activation to completion. These data bring clarity to a confused field, establish the significance of complement in the pathology of progressive MS and provide an evidence base for targeting complement in diagnosis, monitoring and therapy of MS.

## Materials and methods

### Cases

Archival blocks comprising 10% formalin-fixed, paraffin-embedded CNS tissue obtained at autopsy from 17 clinically and neuropathologically validated MS cases and 16 controls, including 9 controls with other CNS inflammatory and non-inflammatory diseases (labelled neurological controls) were used in this study (patient and control details in Table 1). Tissue was obtained from cerebral cortex from all cases and controls, and from spinal cord from 11 cases and 8 controls. MS and non-neurological control tissue was provided by the UK MS Tissue Bank, Imperial College London. Neuroinflammatory disease control tissue was from the Thomas Willis Brain Bank, Oxford University and Brains for Dementia Research, Brain Bank, IOP, King’s College London. In each case clinical and demographic details and interval between death and tissue collection were provided. Ethical approval was gained from the South East Wales Research Ethics Committee (10/WSE02/4).

**Table 1:** **Patient clinical characteristics details**

Case number	Disease status	Age at death	Sex	Death to TPT	DD	Cause of death
**MS055**	SPMS	47	F	15	32	Pneumonia
**MS058**	SPMS	51	F	15	21	MS
**MS061**	PRMS	56	F	6	>15	Adenocarcinoma
**MS064**	SPMS	66	M	56	28	Pneumonia
**MS128**	SPMS	78	F	22	50	Pneumonia
**MS179**	SPMS	70	F	20	25	Pneumonia
**MS306**	SPMS	78	M	17	42	Clostridium difficile diarrhoea
**MS307**	SPMS	55	M	19	20	MS
**MS312**	SPMS	68	F	24	23	Urinary sepsis
**MS372**	SPMS	82	F	7	46	Pneumonia
**MS383**	PPMS	42	M	17	8	Pneumonia
**MS24**	PPMS	71	F	96	22	Pneumonia
**MS160**	SPMS	44	F	18	16	Pneumonia
**MS225**	SPMS	79	F	23	32	MS, Diabetes mellitus, deep vein thrombosis
**MS230**	SPMS	42	F	31	19	MS
**MS234**	SPMS	39	F	15	15	Pulmonary embolism/Pneumonia
**MS377**	SPMS	50	F	22	23	Pneumonia
**C21**	C	91	F	127	N/A	Pneumonia
**C29**	C	71	M	33	N/A	Abdominal haemorrhage
**C23**	NC	93	F	51	N/A	Alzheimer’s disease
**C36**	C	68	M	30	N/A	Cryptogenic fibrosing alveolitis
**C37**	C	84	M	5	N/A	Pneumonia, bladder cancer
**C38**	C	85	M	27	N/A	Ischaemic heart disease
**C39**	C	82	M	21	N/A	Myelodysplastic syndrome
**C40**	NC	86	F	29	N/A	Acute cortical ischemic infarction
**C41**	C	54	M	20	N/A	Lung cancer
**NP7407-2**	NC	93	F	24	N/A	Acute cortical ischemic infarction
**1228/91-b**	NC	88	F	24	N/A	Acute cortical ischemic infarction
**A140_07**	NC	81	F	17	N/A	Alzheimer’s disease
**A173_08**	NC	85	F	30	N/A	Alzheimer’s disease
**A192_2**	NC	96	F	19	N/A	Alzheimer’s disease
**B5046-15**	NC	42	M	72	N/A	Herpes simplex encephalitis
**B5125-9**	NC	54	M	96	N/A	Herpes simplex encephalitis

SPMS: secondary progressive MS; PRMS: progressive relapsing MS; PPMS: primary progressive MS; NC: Neurological Control; C: non-neurological control with no known history of neurological disease; F: female; M: male; TPT: Tissue preservation time in hours; DD: disease duration.

### Histological staining and lesion characterisation

Paraffin wax tissue sections 4 microns in thickness were cut and stained with Luxol fast blue (LFB) to assess presence of myelin and identify areas of demyelinated plaque, peri-plaque and normal-appearing WM. This allowed identification of lymphocytes, plasma cells and foamy macrophages containing myelin degradation products (also positive with anti-MOG staining). Areas selected as normal-appearing WM were at least 1 cm away from the edge of the demyelinating plaque. Cortical grey matter (GM) was identified adjacent to and sometimes continuous with the peri-plaque; the GM areas showed neither meningeal inflammation nor perivascular inflammatory cells.

**Figure 1:**
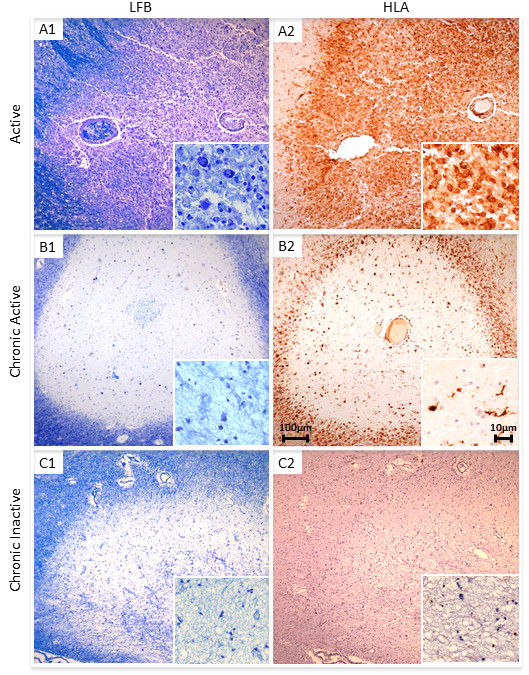
**Plaque morphology in MS cases.** Paraffin wax sections stained with LFB **(A1, B1 and C1)** and anti-HLA DR **(A2, B2 and C2)** showing an active (**A1-A2**; case MS160_S3), chronic active (**B1-B2**; case MS225_S13) and chronic inactive plaque (**C1-C2**; case MS61_B). The active plaque shows profound inflammation with anti-HLA-DR immunopositive microglia (**A2** and inset) and foamy macrophages containing myelin degradation products demonstrated using LFB (inset in **A1**). The chronic active plaque shows little inflammation in the centre of the plaque (insets in **B1** and **B2**) but abundant anti-HLA-DR immunopositive microglial at the plaque border **(B2)**. The chronic inactive plaque shows an immunologically silent lesion centre (inset in **C1** and **C2**) and a well demarcated lesion border showing only mild inflammation (**C2** and inset) Scale bars are shown in **B2** and are applicable for all plates and inserts.

Anti-HLA-DR antibody was used to identify microglia and anti-MOG antibody to detect myelin. MS lesions were classified as either classical active (profound inflammation with microglia and macrophages containing myelin degradation products) or slowly expanding (central profound axonal loss with loss of macrophages, with a rim of activated microglia and moderate inflammation) [14]. Slowly expanding lesions were further subdivided into either chronic active (residual plaque centre inflammation and profound microglial inflammation at the plaque border) or chronic inactive (immunologically silent lesion centre with a well demarcated lesion border showing only mild inflammation) (examples shown in Figure 1) [14]. Spinal cord sections were also analysed for plaque subtype according to this classification. To ensure accuracy of lesion classification, all sections were assessed by three independent trained observers (JWN; OWH; GI); inter-observer concordance was 100%.

### Immunohistochemistry

Paraffin wax sections were de-paraffinated using xylol and rehydrated in serial aqueous dilutions of ethanol (100%, 96%, 70%, 0%). For antigen retrieval, sections were either heated in citraconic anhydride (pH 7.4; 45 mins at 100°C) or immersed in protein kinase 20 μg/ml (20 mins at 37°C; for primary antibody B7 only). Prior to staining, sections were blocked in 10% normal goat serum. To ensure optimal consistency, staining was performed using an autostainer (DAKO EnVision™ + System). Endogenous peroxidase was blocked by 10 mins incubation in 3% H_2_O_2_. Primary antibodies, including antigen target, clone, concentration, dilution and source, are listed in Additional file 1: Table S1. Appropriate secondary antibodies were from Dako and DAB chromogen was used for visualisation.

Double labelling was performed with two peroxidase steps according to the manufacturer’s recommended protocols (http://www.vectorlabs.com, A guide to multiple antigen labelling); blocked sections were incubated for 1 hour with the first primary antibody followed by 30 mins with the appropriate peroxidase-labelled secondary antibody and then stained using Vector SG peroxidase substrate kit (Vector Laboratories, http://www.vectorlabs.com). Sections were subsequently blocked for 10 mins with 10% normal goat serum, incubated for 1 hour with the second primary antibody and for 30 mins with the peroxidase-labelled secondary antibody which was visualised with DAB chromogen. In this system the peroxidase substrate yields a grey stain and the DAB label a brown stain, enabling the two primary antibodies to be visualised on the same slide in different colours. The sections were dehydrated prior to permanent mounting in DPX.

For immunofluorescence, sections were stained sequentially for complement and cell specific markers at room temperature, according to published protocols [15]. When two monoclonal antibodies of different IgG isotypes were tested (HLA-DR (IgG1) and complement activation product iC3b (IgG2a), for example), then subtype selective goat anti-mouse IgG1-Cyanine 2 conjugate (Stratech Scientific, Newmarket, UK), and biotin-linked goat anti-mouse IgG2a (Abcam, Cambridge, UK) and streptavidin- Alexa 594 (Life Technologies, Paisley, UK), were applied sequentially. Negative controls were run in parallel, and involved omission of one or both primary antibodies and/or inclusion of an irrelevant isotype control antibody. All such controls were devoid of staining. Following incubations with the secondary antibodies, all sections were counterstained with 4′,6-Diamidino-2-phenylindole dihydrochloride (DAPI; Sigma), quenched in 0.1% w/vol Sudan black B (VWR International), rinsed in deionised water and coverslipped with Vectashield aqueous mountant (Vector Labs, Peterborough, UK).

Sections were analysed on a Zeiss Axio Imager 2 under epifluorescence and images captured using a Zeiss AxioCam MRm camera or by confocal laser scanning microscopy with Zeiss LSM-710 and inverted Axio Observer microscope with oil-immersion 40X and 63X objectives. All images were analyzed off-line using ImageJ (http://rsb.info.nih.gov/ij/) and prepared in Photoshop CS2 (Adobe Systems, Uxbridge, UK) for publication.

Positive control tissue sections for testing the anti-complement antibodies comprised arrayed fixed tissue sections [16] from renal biopsies taken from glomerulonephritis patients and myocardium from a patient with myocarditis due to adenovirus; all antibodies positively labelled these sections using the described protocols (Additional file 2: Figure S1).

### Quantitative analysis

All stained sections were digitally scanned at x40 magnification of the objective lens (0.037 mm^2^/field). In each section, areas representative of plaque (P), peri-plaque (PP), WM and GM were identified and five different fields from each area were selected manually for further analysis. For quantification, the selected fields were overlaid with a grid and positively labelled cells within the grid for each of the individual antibodies were manually counted. Values were expressed as the number of positively labelled cells per mm^2^. In order to verify data, inter-rater agreement was assessed between two independent trained observers (GI; SL) over 30 scanned fields from different sections, patients and primary antibodies; inter-observer correlation coefficient was 0.99. Non-cellular interstitial staining and myelin staining was classified as either negative (-), weakly positive (+), moderately positive (++) or strongly positive (+++) based on criteria established in house [17].

### Statistical analysis

Data analysis was performed using Statistical package for Social Sciences version 20 (SPSS, Chicago, USA). Data were not normally distributed and therefore were analysed using non-parametric tests. MS patients were compared to normal and neurological control groups using the Kruskal-Wallis Test for independent values. Where positive, subgroups were analysed using the Mann–Whitney U test. Differences between MS disease subgroups (P, PP, WM, GM) were analysed using Freidman’s ANOVA and where positive, post-hoc procedures were performed using the Wilcoxon Signed Ranks Test. Differences between active, chronic active and chronic inactive plaques were analysed using the Kruskal-Wallis Test for independent values. Where positive, subgroups were analysed using the Mann–Whitney U test. For most tests a P value of <0.05 was considered significant. For subgroup analysis to compensate for the inflation of type I errors due to multiple testing, a p value of <0.025 was taken as significant (0.05 divided by the number of tests).

## Results

### Characteristics of MS lesions studied

**Figure 2:**
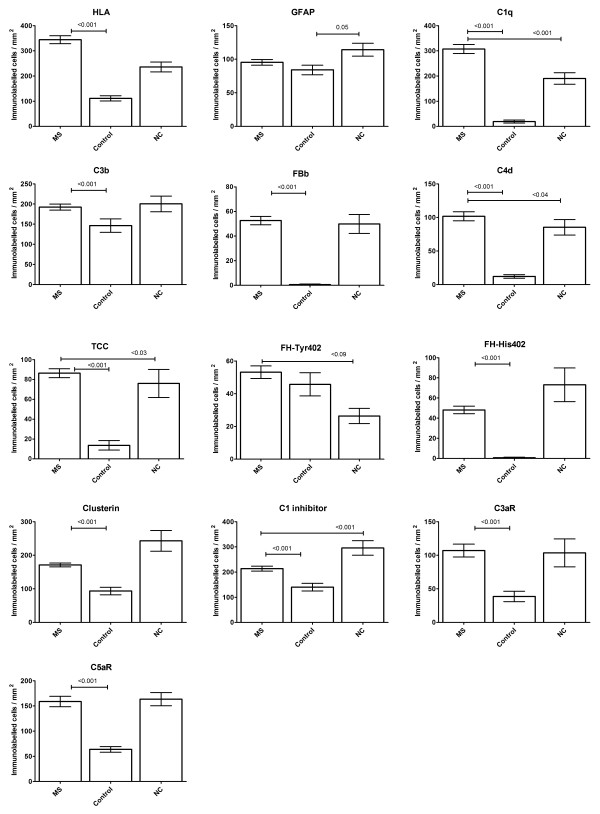
**Quantitation of cell immunolabelling.** Quantitative cellular immunolabelling in brain and spinal cord tissue from multiple sclerosis (MS; 17 cases with 690 areas examined from 42 sections), non-neurological controls (Controls; 7 cases with 140 areas examined from 14 sections) and neurological controls (NC; 9 cases with 110 areas examined from 11 sections). Groups show total mean values from all areas examined +/_ standard error. Significant results are shown by p value examining differences between the MS group and controls or neurological controls; differences between the two control groups were not included for reasons of clarity.

**Figure 3:**
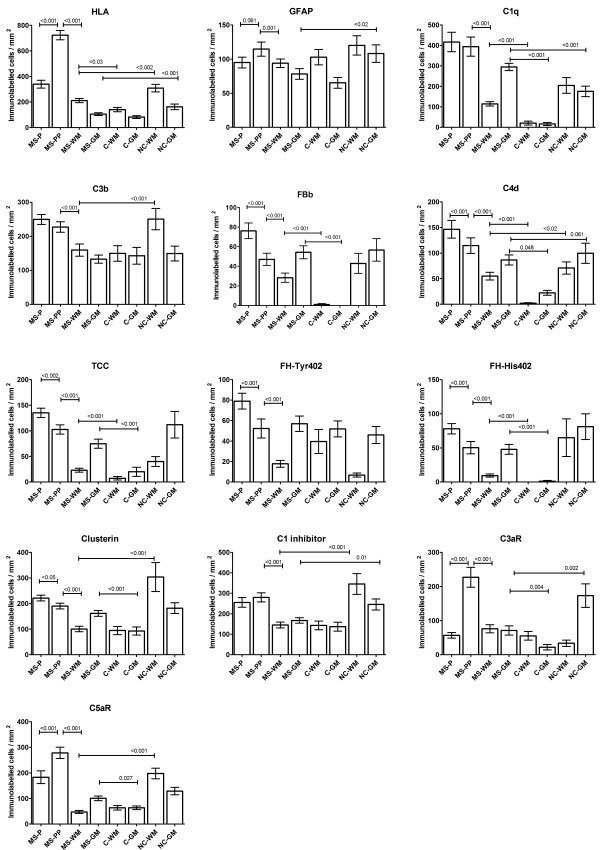
**Quantitative immunolabelling of cells from different tissue areas.** Quantitative cellular immunolabelling in different tissue areas from sections of brain and spinal cord tissue from multiple sclerosis (MS; 42 sections from 17 cases with 175 areas examined within the plaque (P), 175 within the peri-plaque (PP), 145 within the white matter (WM) and 195 within the grey matter (GM)), non-neurological controls (C; 14 sections from 7 cases with 70 areas examined from both WM and GM) and neurological controls (NC; 11 sections from 9 cases with 55 areas examined from both WM and GM). Groups show mean values +/_ standard error. Significant results are shown by p value examining differences between the MS group and controls or neurological controls for both WM and GM; differences between the control groups were not included for reasons of clarity. Within the MS group, differences were analysed between P and PP/PP and WM.

All cases examined were from patients with progressive MS, most with long-standing disease (mean disease duration 26 +/- 12 yrs, mean age at death 59 +/- 15 yrs) (Table 1). Both control groups had higher mean age at death (non-neurological controls 76 +/- 13 yrs, p = 0.021; neurological controls 80 +/- 19 yrs, p = 0.003). There were no significant differences between the time to tissue preservation (TTP) in each group (MS 24.88 +/- 21.36 hrs; non-neurological controls 37.57 +/- 40.48 hrs, p = 0.317; neurological controls 40.22 +/- 27.35 hrs, p = 0.190). MS lesions were classified depending on lesion activity. Of the 35 plaques examined, 8 were classified as active and 27 as slowly expanding, 9 of the chronic active and 18 of the chronic inactive type (Figure 1). An additional seven MS sections examined comprised only WM and GM with no plaque. MS sections were almost devoid of lymphocytes or plasma cells with only a few T or B cells identified within the blood vessel wall and perivascular space and an occasional T cell observed within the plaques of active lesions (Additional file 2: Figure S1). CNPase positive oligodendrocytes were universally absent from all plaques examined. In MS WM and GM, astrocyte cell counts were not significantly different from non-neurological or neurological controls (Figures 2 and 3); however microglial cell counts were significantly higher in MS WM than non-neurological control WM, but higher still in the WM and GM of the neurological control group (this being the area of pathology in this group).

### Complement immunolabelling is ubiquitous in MS lesions

Complement protein immunolabelling was observed in all sections from MS cases to some degree; in contrast, immunolabelling was completely absent in two of seven non-neurological controls and minimal, in the other five. Staining in non-neurological controls was largely restricted to background extracellular immunolabelling, except for antibodies against C3b, clusterin, fH-Tyr402 and C1inhibitor (C1inh) which significantly stained cells in some controls (Figure 2). One of nine neurological controls (an Alzheimer’s disease case) showed no complement staining, but for the other neurological controls, staining for most complement proteins was seen in a similar number of cases to that in MS; the exception was C1q staining which was significantly higher in MS tissue (Figure 2). Paraffin wax sections from kidney tissue with glomerulonephritis and myocardium with viral myocarditis showed immunolabelling in relevant areas for all antibodies used (Additional file 2: Figure S1). Sections stained with isotope control antibodies (IgG1 or IgG2) were consistently negative in both MS cases and controls (Additional file 2: Figure S1).

### Complement immunolabelling in MS is predominantly cell-associated

The majority of observed complement immunolabelling in MS cases was cell-associated, either surface or within the cell (not distinguishable in immunohistochemistry), while cell immunolabelling in non-neurological controls was weaker and predominantly nuclear (Additional file 2: Figure S1). Cell immunolabelling in the neurological control group was marked, especially in areas of inflammation and obvious disease pathology, for example, in and around Alzheimer’s plaques (Additional file 2: Figure S1).

**Figure 4:**
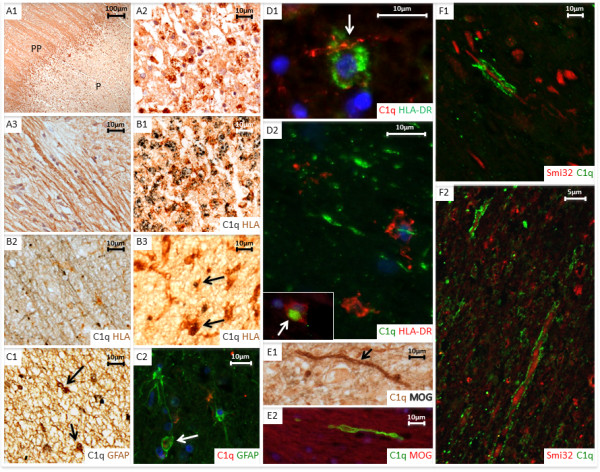
**Cell associated C1q complement staining.** Paraffin wax sections **A1**, **A2** and **A3** (case MS160_S3, active case). **A1** shows immunolabelling with anti-C1q in plaque (P) and peri-plaque (PP) areas. **A2** shows C1q immunopositive debris (brown) within foamy macrophages localized in the centre of an active plaque. **A3** shows C1q immunolabelled on myelin. **B1** paraffin wax section double immunolabelled with anti-C1q (grey) and anti-HLA-DR (brown) confirms C1q immunopositive debris located within HLA positive foamy macrophages cells; (case MS160_S3, active plaque). **B2** and **B3** also show paraffin wax sections with immune double staining of anti-C1q and anti-HLA-DR (case MS230_s2, chronic active plaque). **B2** shows HLA-DR positive cells (brown) closely associated with C1q positive myelin (grey) within the white matter. **B3** shows co-localisation of C1q on HLA-DR positive microglia (arrows) in the peri-plaque. **C1** (case MS372_22, chronic active plaque) and **C2** (case MS230_S2, chronic active plaque, stained using immunofluorescence (IFC)) show double labelling for C1q and GFAP, with colocalisation of C1q (grey) and GFAP (brown) in some but not all cells (insert in **C1** and arrow in **C2** highlighting colocalisation). IFC in figure **D1** (case MS377_S2, active plaque) and **D2** (case MS160_S1, active plaque) demonstrate anti-HLA-DR positive macrophages closely associated with C1q positive myelin (arrow). Inset in **D2** shows C1q immunopositive debris within an HLA-DR immunolabelled macrophage (arrow). **E1** shows a myelin sheath at edge of an active plaque staining with anti-MOG (grey) and C1q (brown) (case MS160_S3, arrow); in the same area **E2** (IFC, case MS160_S3) shows a myelin sheath with positive anti-C1q immunolabelling. Figures **F1** (MS160_S3/1; chronic active plaque) and **F2** (MS336_1; active plaque), captured with confocal laser scanning microscopy, show disrupted myelin, immuno-positive for C1q associated with anti-SMI-32 immunopositive non-phosphorylated axon profiles in chronic and active areas. Scale bars are shown for each plate.

**Figure 5:**
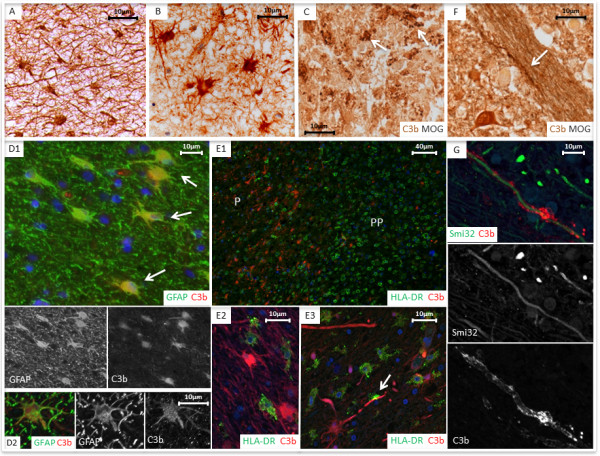
**Cell associated C3b complement staining.** Paraffin wax section of active peri-plaque regions (**A**, case MS377_S2; **B**, case MS383_21) immunolabelled with anti-C3b showing cells with astrocytic morphology. Myelin degradation debris (arrows) within macrophages colabelled with anti-C3b (brown) and MOG (grey) are shown within the centre of an active plaque (**C**, MS160_S3). Immunofluorescent staining: **D1** (case MS 225_S13, chronic active plaque) and **D2** (case 377_S2, active plaque) showing colabelling of C3b (red) and GFAP + astrocytes (green) in the peri-plaque white matter. **E1** (case MS225_S13, chronic active plaque; plaque (P) and peri-plaque (PP)), **E2** and **E3** (both case MS377_S2, active plaque) show zones of HLA-DR macrophages/microglia and an adjacent area of predominantly astrocytic and axonal C3b immunolabelled structures. In figure **E3**, note the close approximation of C3b positive axon (arrow) and HLA positive microglial cell. **F** shows an anti-C3b positive myelin strand (brown, arrow) co-localised with MOG (grey) on an axon bundle within striatum (case MS160_S3, active plaque). Immunofluorescent staining **(G)**, showing anti-C3b immunopositive myelin (red) co-localised with an anti-SMI-32 (green), immunopositive non-phosphorylated axon at the edge of a chronic active white matter lesion (case MS160_S3, active plaque). Scale bars are shown for each slide.

Cell complement immunolabelling within the plaque and peri-plaque areas in MS sections was consistently associated with cells morphologically resembling astrocytes for all complement proteins tested except the anaphylatoxin receptors C3aR and C5aR (Figures 4, 5 and Additional file 3: Figure S2). Double immunolabelling confirmed this observation; C1q and C3b co-localized with GFAP + reactive astrocytes within the plaque and peri-plaque (C3b in this manuscript refers to immunolabelling with the C3b/iC3b-specific monoclonal antibody C3/30) (Figures 4 and 5). C1q and C3b stained reactive astrocytes were often in close proximity to clusters of HLA-positive microglia with activated morphology and macrophages containing complement-stained debris, seen especially in active plaque centres (Figures 4 and 5). C1q also co-localized with HLA + activated microglia, contributing to the increased cell-associated C1q immunolabelling in the peri-plaque (Figures 3 and 4); these cells were also positive for anaphylatoxin receptors C3aR and C5aR (Additional file 3: Figure S2). GFAP positive astrocytes within the GM did not stain for complement; indeed, complement immunolabelling of all complement antibodies within the GM in MS and controls was mainly limited to neurones (Additional file 4: Figure S3).

The number of cells immunolabelling for each complement protein was counted in all fields and compared, MS with controls, and within the MS sample, plaque, peri-plaque, WM and GM (Figures 2 and 3). Cellular immunolabelling of all complement antibodies in the neurological control group was restricted to cells morphologically resembling microglia within WM and neurones within GM. No complement component immunolabelling was observed on astrocytes in either normal or neurological control groups. This finding was confirmed with double labelling experiments (Additional file 2: Figure S1).

### Complement activation markers are characteristic of MS

Cellular and non-cellular immunolabelling for the classical pathway protein C1q was strongly positive in both MS and neurological control groups; 100% of MS sections were stained for C1q compared to 77.8% of neurological control sections, while 10% (p < 0.001) of normal control sections showed weak C1q staining (Additional file 5: Figure S4). There were significantly more positively stained cells in MS sections compared to both non-neurological and neurological controls (p < 0.001; Figure 2). In MS sections, C1q immunolabelling was found on cells morphologically resembling both astrocytes and microglia, most abundantly in the plaque and peri-plaque areas but also in WM; in GM, C1q immunolabelling of neurones was seen (Figures 3 and 4).

The majority of MS sections were positive for the classical pathway activation fragment C4d (Additional file 5: Figure S4); despite some cellular immunolabelling of C4d in control sections, cell immunolabelling for C4d in MS sections was markedly higher than in non-neurological controls (p < 0.001, Figure 2), and was most abundant in peri-plaque and plaque and minimal in WM (Figure 3). In GM, some cell-associated (predominantly neuronal) C4d immunolabelling, was present in all groups (Additional file 4: Figure S3).

Alternative pathway activation fragment Bb (Bb) was positive in 59.5% of MS sections compared to 7.1% (p < 0.001) of non-neurological controls and 54.5% of neurological controls (Additional file 5: Figure S4), indicating ongoing activation of the alternative pathway in MS. Immunolabelling of cells morphologically resembling astrocytes, was most abundant within the peri-plaque and plaque (p < 0.001; Figure 3), although Bb-immunostained cells were present at increased levels in MS compared to non-neurological controls in both WM and GM (p < 0.001; Figure 3), suggesting that alternative pathway activation occurred throughout the brain in MS, not just within the plaque core.

Cell immunolabelling for C3b/iC3b was detected in almost all MS sections, but was also present in neurological controls and weakly in some non-neurological controls (90.5% MS, 50% non-neurological controls (p < 0.001), 81.8% neurological controls; Additional file 5: Figure S4); numbers of positively stained cells/mm^2^ in MS sections were significantly higher compared to non-neurological controls (p < 0.001; Figure 2); staining was strongest in plaque and peri-plaque (Figure 3). These findings were confirmed in three MS cases (MS 55, MS 61 and MS 64) using another mAb specific for iC3b (A209, data not shown). Quantified cellular immunolabelling of these two C3b/iC3b mAbs (C3/30 and A209) showed an intraclass correlation coefficient of 0.85 (95% CI 0.76-0.91, p < 0.001).

Terminal pathway activation assessed using a TCC-specific mAb was positive in 71.4% of MS sections compared with 7.1% of non-neurological controls (p < 0.001) and 54% of neurological controls (Additional file 5: Figure S4), with significantly more positively stained cells in MS cases compared to non-neurological controls (p < 0.001, Figure 2). Immunolabelling for TCC was strongest in the plaque and peri-plaque but was also present in WM and GM where TCC immunolabelled cells (in MS groups morphologically resembling astrocytes in the WM (Additional file 4: Figure S3) and neurones in the GM) were more frequent than in non-neurological controls (p < 0.001 for both, Figure 3). Of note, all MS sections that contained TCC immunopositive cells also showed comparable positive immunolabelling for C3b; however, some sections positive for C3b showed weak or absent TCC staining.

### Cell expression of complement regulators and receptors is altered in MS

To inform speculation regarding consequences of local complement activation on CNS cells in MS, sections were stained with mAb specific for the key plasma-derived complement regulators C1inh, factor H (fH) and clusterin, and the anaphylatoxin receptors C3aR and C5aR. This selection was pragmatic and dictated by availability of mAb (in house or commercial) that were suitable for use in formalin fixed tissue. Expression of both classical pathway regulator C1inh and terminal pathway regulator clusterin was found in most MS sections, strongest in astrocytes in the plaque and peri-plaque area (Figure 3 and Additional file 4: Figure S3 and Additional file 5: Figure S4). Although cell expression of both C1inh and clusterin was stronger and more frequent in MS compared to non-neurological controls (Figure 2), there was background WM and GM immunolabelling in both MS and control sections (Figure 3).

Expression of the alternative pathway regulator fH was assessed using mAb specific for the Tyr402His polymorphic variants (described in [18]). Cellular immunolabelling for either or both variants was seen in 13 of 17 MS cases with higher numbers of positively immunolabelled cells in the plaque and peri-plaque areas compared to the WM (p < 0.001; Figure 3); cellular immunolabelling for the two variants was positive in each case. Eight of nine neurological controls showed cell immunolabelling for Tyr402 and seven of these were also His402 positive. Of the seven non-neurological controls, five were Tyr402 positive and one was also positive for His402 (Additional file 5: Figure S4). In MS sections, 26 of 42 showed immunolabelling for either or both variants with high numbers of positively immunolabelled cells in the plaque and peri-plaque areas compared to the WM (p < 0.001; Figure 3); of the 26 positive sections, 6 stained only for Tyr402, 17 stained for both variants and 3 stained only for His 402. In normal control sections, 9 of 14 sections showed immunolabelling, 8 of these stained for Tyr402 only and one for both variants. In neurological control sections, 6 of 11 sections showed immunolabelling, 3 of these stained for Tyr402 only and 3 for both variants (Additional file 5: Figure S4).

Cellular immunolabelling of the anaphylatoxin receptors C3aR and C5aR was restricted to microglia with morphology compatible with activation. Immunolabelling for C3aR was observed in 90% of MS sections and 40% of non-neurological controls (p = 0.004, s0al Figure 4) whereas C5aR was observed in the majority of MS and control sections, although the number of immunolabelled cells in MS cases and neurological controls was almost 3-fold higher compared to non-neurological controls (p < 0.001 for both C3aR and C5aR; Figure 2). Cell immunolabelling for C3aR and C5aR in MS sections was highest in the peri-plaque compared to plaque and WM (p < 0.001; Figure 3), with predominant co-localization with HLA positive microglia within the peri-plaque areas (Additional file 3: Figure S2). C3aR immunolabelling was also observed on the cerebral vessel endothelium and smooth muscle cells within the vessel wall in MS tissue. C3aR and C5aR immunolabelling in WM and GM was weak and not different in MS compared to non-neurological control sections; in contrast in neurological control WM and, even more markedly, GM, strong microglial staining for C3aR and C5aR was consistently found (Figure 3).

### Complement immunolabelling of myelin is characteristic of MS

Immunolabelling for one or more complement protein was present on myelin, intact or with a fragmented or disrupted appearance in 64.3% of MS sections compared to 36.4% of neurological control sections and 28.6% of non-neurological control sections (comparison of myelin immunolabelling in MS versus non-neurological controls; p = 0.035, Additional file 5: Figure S4). Complement immunolabelling of myelin in MS was predominantly observed in peri-plaque areas, with some immunolabelling of fragmented myelin within plaques (Figures 4 and 5 and Additional file 4: Figure S3). Of the MS sections, 14.3% showed positive myelin immunolabelling for C1q, 7.1% for Bb, 19% for C3b, but none were stained for TCC. Of the regulators, 26.2% of MS sections showed positive myelin immunolabelling for C1inh and 9.5% for clusterin; myelin staining for fH variants was remarkable in that staining for the less common fH-His402 variant was almost twice as frequent as for the more common fH-Tyr402 variant (31% versus 16.7% of sections and seven compared to five out of 14 cases).

Myelin staining for regulators was minimal in controls except for low level C1inh staining in non-neurological controls (one case) and staining for clusterin (one case) and fH-His402 (two cases) in neurological controls; however, because of the broad variability between sections for myelin immunolabelling, only fH-His402 was significantly different from controls (Additional file 5: Figure S4). In MS sections, myelin fragments immunopositive for complement proteins were frequently present at the lesion edge, associated with both HLA-positive microglia and SMI32-positive stressed/damaged axons (Figures 4 and 5). These features were not observed in control sections.

### Complement immunolabelling marks active plaques

Significantly more immunolabelling of complement proteins, activation products and regulators was observed in the plaque and peri-plaque areas of active and chronic active compared to inactive plaques (Additional file 6: Figure S5). Overall there was little difference in complement immunolabelling observed between the active and chronic active plaques except for two of the regulators (fH-His 402 and clusterin) where immunolabelled cells were observed more frequently in chronic active plaques (Additional file 6: Figure S5).

Immunolabelling of C3aR was minimal in the active plaque centres and peri-plaque areas, but seen much more frequently in the inactive peri-plaque; where although few microglia were observed, the majority of these showed C3aR immunolabelling. C5aR immunolabelling was observed equally in all plaque types (Additional file 6: Figure S5).

All of the plaques in spinal cord sections examined were inactive (making up ten of the 18 inactive plaques examined). Immunolabelling for the majority of complement antibodies was similar to but significantly lower in the spinal cord sections compared with brain sections, except for the anaphylatoxin receptors in which immunolabelled cells were observed more frequently in cord (data not shown).

## Discussion

There is on-going debate surrounding the contribution of inflammation to the relentless axonal degeneration seen in MS patients, clinically most apparent in progressive disease; absence of immune cells and lack of efficacy of anti-inflammatories has led some to postulate a complex and as yet undefined neurodegenerative process to explain the relentless brain atrophy [19]. We show that complement proteins, activation products, regulators and receptors are markers of CNS innate immune activation, present in active plaques but persisting even in late stage disease when plaques become inactive, indicating that synthesis, activation and regulation of complement are all on-going in plaque and peri-plaque areas of brain and spinal cord in late-stage MS.

In chronic active and inactive plaques, complement markers occurred in the absence of other inflammation markers, including lymphocytes, plasma cells and foamy macrophages; demonstrating that progression of inflammation in MS CNS does not rely on infiltrating cells; once initiated, inflammation can be driven by innate immune mechanisms such as complement. These findings support our previous studies showing systemic complement activation and regulation in MS throughout the disease course [20]; in particular, increased serum fH levels were specific to progressive disease and predicted relapse [18], suggesting an active role in late stage disease.

The profile of complement immunolabelled cells was similar in patients with active and chronic active plaques but markedly different in patients with other neuroinflammatory diseases - encephalitis, Alzheimer’s disease and ischaemic infarction. In MS, complement staining was predominantly associated with GFAP + astrocytes in plaque and peri-plaque areas; in contrast, in neurological controls, cellular complement staining associated with microglia in WM and neurones in GM. In encephalitis and ischaemic infarction cases, the time from pathological insult to death was short in comparison to Alzheimer’s disease and MS where complement expression and activation is maintained over years. In ischaemic infarction there is catastrophic blood brain barrier (BBB) disruption, facilitating entry of complement; in MS, Alzheimer’s disease and encephalitis where the degree of BBB disruption is uncertain, local synthesis may be an important contributor and it is known that glial cells can produce all complement components needed for terminal pathway activation [21].

Evidence implicating the classical pathway in MS pathogenesis was described over thirty years ago. Immune complexes were demonstrated in MS CSF and brain with associated binding of C1q and classical pathway activation [22]; in parallel, it was shown that oligoclonal immunoglobulins were present in CSF in most MS patients [23], the result of intrathecal synthesis [24]. Even in the absence of antibody, the classical pathway can be triggered by C1q directly binding damaged myelin [25]. Here we confirm that C1q is a powerful disease marker, present in all MS cases and minimal in controls. C1q immunolocalized to reactive astrocytes, microglia and myelin in peri-plaque and plaque areas; immunolabelling does not distinguish between protein *on* and *in* cells - it is entirely possible that astrocyte staining for C1q and other complement proteins represents biosynthesis, especially in inactive chronic lesions where BBB breakdown is minimal. In contrast, astrocyte- and myelin-associated C3- and C4-derived activation fragments and TCC in MS brain is strong evidence of ongoing classical pathway activation and implies that at least some of the C1q is on the astrocyte surface and activating. The alternative pathway marker Bb was positive in the majority of MS cases, suggesting that amplification through this pathway was a significant contributor.

Complement regulators assessed in this study showed increased immunolabelling in plaque and peri-plaque areas indicating the ongoing capacity for local complement inhibition throughout MS disease course. In light of our published finding that the relative plasma levels of the variants of regulator fH alter in progressive MS [18], the Tyr402His polymorphic variants (minor allele frequency 0.3) were separately measured; remarkably, the relative staining of Tyr and His variants was markedly different between MS cases and controls, suggesting that there were qualitative differences in fH variant expression and/or deposition between cases and controls with more frequent cell-associated fH, and in particular the His402 variant, in MS. The functional consequences and relevance to disease progression of this remain to be ascertained; however, in age-related macular degeneration (AMD), differential binding of the fH-Tyr402His variants in retina has been demonstrated and proposed as the mechanism by which fH-His402 is risk for AMD [26]. Of note, we previously demonstrated increased plasma levels of fH and altered Tyr402His ratio in progressive MS with over-representation of His402 in heterozygous patients [18].

Our data demonstrate considerable heterogeneity of complement immunolabelling in MS cases. Pathological heterogeneity and heterogeneity of complement staining in MS lesions was described by Lucchinetti and colleagues; they proposed four distinct patterns of MS lesions with only 50% of patients showing complement immunolabelling, limited in their study to TCC immunolabelling using the mAb B7, also used in our work [10, 27]. Others have contested this, using a wider range of complement markers and reporting consistent complement immunolabelling in all lesions and cases examined [11]. Our data agree in part with the findings of Lucchineti in that 71% of sections were TCC+; however, all were C1q +, most C3b/iC3b + and all TCC + sections were strongly C3b/iC3b+. Together these data indicate that complement staining is a ubiquitous feature of MS lesions but the pattern of staining is variable. We suggest that TCC is a reliable marker for complement activation in MS; its near-complete absence in controls supports its use in isolation as a marker. In our hands, very few lesions were negative for TCC and C3b/iC3b and these were C1q+, which may also be a useful marker despite its presence in control tissues.

The predominant staining of reactive astrocytes for complement activation products including iC3b and TCC has not previously been described in MS and was disease-specific; complement activation marker staining in neurological controls was extracellular and on microglia/macrophages in the tissue. The data suggest that this astrocyte-restricted pattern of complement staining is specific to demyelinating disease. Complement-mediated astrocytic impairment was recently described in neuromyelitis optica [28]; our findings imply that this may also be a feature of progressive MS. Complement-stained astrocytes were often co-localised with clusters of activated microglia, a finding that has previously been reported in MS and linked to complement priming of microglia contributing to CNS inflammation [29]. The notion of astrocytes contributing to pathological process in MS by enhancement of immune response and disruption of myelin has recently been raised in a number of studies [30, 31].

Immunolabelling of disrupted myelin in MS lesions, previously cited as evidence of complement-mediated pathology, was limited and variable in this study. Breij et al. [11] described myelin staining for C3d but not TCC in active lesions. Myelin staining for complement proteins has been suggested to be a non-specific feature [12]; however, using double immunolabelling we found complement markers co-localized in areas of degenerating myelin and damaged axons in MS sections (Figures 4 and 5), implicating complement in axonal and myelin injury in and around active and chronic plaques. Absence of TCC may reflect efficient regulation of terminal pathway activation at these sites or an inability of TCC/MAC to intercalate into myelin. Clusters of activated microglia were frequently found in close apposition to damaged myelin, likely representing iC3b/CR3-mediated opsonic clearance.

C3aR and C5aR were expressed on microglia in and around the plaque, a finding previously reported in acute plaques [32, 33], but not in chronic active and inactive slowly expanding lesions studied here; indeed, numbers of C3aR/C5aR-positive microglia were higher in chronic/inactive lesions than in the few active plaques observed. The abundance of C3aR/C5aR-positive microglia in the peri-plaque suggests a capacity for locally generated C3a and C5a to recruit and activate microglia even in late lesions.

## Conclusions

This study supports the concept of progressive inflammation in late stage disease, driven by complement and contributing to cell, axon and myelin damage, microglial activation and chemoattraction. Selective recruitment of complement regulators to sites of activation may give further clues to underlying pathology. The findings support the validity of measurements of serum complement activation and of imaging complement activation as ways of measuring and monitoring MS disease activity in vivo. Imaging complement activation using labelled antibodies or ligands to target activation products is a rapidly evolving concept that might find particular relevance in MS and other CNS diseases [34, 35]. Our data show that C1q, iC3b and TCC all present viable imaging targets in MS. Identification of complement activation through a combination of imaging and plasma markers might identify those patients most likely to benefit from the growing list of anti-complement therapies.

## Electronic supplementary material

Additional file 1: Table S1: Antibodies used for immunohistochemistry. (PDF 95 KB)

Additional file 2: Figure S1: Control sections. Figures showing examples of complement immunolabelling in paraffin wax sections from white matter from cases of acute and chronic neurological inflammation and normal control white matter immunostained with antibodies to C3b, C1q and C1 inhibitor. Alzheimer’s disease (AD), cases A192 and A173 (plates A1-A5), shows minimal immunolabelling of complement except within the amyloid plaque (inset A1). Herpes Simplex Virus encephalitis (HSV), cases B5046-15 and B5125-9 (B1-5) shows more cellular and non-cellular immunolabelling of complement. Cellular immunolabelling of C3b (C1), C1q (C2) and C1inh (C3) is demonstrated in white matter of a case of ischaemic stroke (case 122891-b) with immunolabelling of macrophages and microglia within the infarcted tissue. Non-neurological control white matter, cases C041 and C038 (D1-5) showed minimal complement immunolabelling. Double labelling in both neurological and non-neurological control cases demonstrated colocalisation of C3b with HLA positive microglia (D4) but not GFAP positive astrocytes (D5). E1 demonstrates positive immunolabelling of C3b within the glomeruli of kidney sections with glomerulonephritis. E2 (C1q) and E3 (C1inh) demonstrate C1q deposition on the cardiac myocardial cells and C1 inh located on the sarcolemma in cardiac myocytes in section of myocardium from patients with myocarditis due to an adenovirus infection. Isotype anti-IgG1 (F, case MS64_B) and anti-IgG2 (not shown) were consistently immunonegative. MS tissue containing an active plaque (G) with a small diameter blood vessel demonstrated immunolabelling of T lymphocytes with in the blood vessel wall and occasionally within the perivascular space; minimal positive immunolabelling for anti -CD3 was is seen in the plaque. Scale bar is shown in A1 and is applicable for plate A, B, C and D. Scale bars for plates E, F and G are shown on each plate. (TIFF 2 MB)

Additional file 3: Figure S2: Complement anaphylatoxin receptors. Paraffin wax sections immunolabelled with anti-C3aR (A1-4) and anti- C5aR (B1-4). Figure A1 (case MS307_20, inactive plaque) and B1 (case MS230_S2, chronic active plaque) shows a low power image of the plaque (P) and peri-plaque (PP) with immunolabelled C3aR (A1) and C5aR (B1) cells. Immunopositive C3aR and C5aR labelled cells are seen predominantly at the lesion edge; C3aR demonstrated in A2 (case MS307_20, inactive plaque) and C5aR in B2 (case MS160_S3/1 chronic active plaque). Both C3aR (A3, case MS312_19, chronic active plaque) and C5aR (B3, case MS307_20, inactive plaque) (grey) were shown to co-localise with HLA-DR + microglia (brown, arrow). No co-localisation was shown with GFAP + astrocytes (brown); C3aR demonstrated in A4 (case MS312_19, chronic active plaque) and C5aR in B4 (case MS307_20, inactive plaque) (grey). Scale bars are shown for each slide. (TIFF 912 KB)

Additional file 4: Figure S3: Complement antibody staining in MS. Plate A demonstrates paraffin wax sections showing anti-C4d immune-positive cells of astrocyte morphology in an active lesion (case MS179_B). Alternative pathway component Bb is shown in plate B on immunolabelled cells within a chronic active plaque (case MS225_S13; arrow indicates an immunolabelled cell of astrocyte morphology). C shows immunolabelling with anti-TCC demonstrating immune-positive cells with astrocyte morphology (arrow). D shows C1q immunopositive neurones in the cortex of case MS55_B. E1 and E2 show immunolabelling with anti-C1 inhibitor demonstrating immune-positive cells with astrocyte (E1, arrow) and microglial-like (E1, dashed arrow) morphology as well as immunolabelling on individual myelin sheaths (E2, arrow) (both case MS160_S1, active plaque). F1 and F2 (F1: case MS64, inactive plaque. F2: case MS128_B, cortex) show anti-clusterin immune-positive cells of astrocyte morphology (F1, arrow) and neurones (F2). Case MS179 is heterozygote for the factor H (fH) Tyr402His polymorphism. Immunolabelling with fh_His402 (fH-H) and fH-Tyr402 (fH-T) demonstrates immune-positive cells within the plaque (G2, fH-Tyr402, arrow) and peri-plaque (G1, fH-His402, arrow and G3, fh-Tyr402) with fragmented myelin shown in the peri-plaque areas. G4 demonstrated neuronal immunolabelling with fH-Tyr402 within the cortex (case MS64_B). Plate H1-H4 show immunolabelling of C1q, clusterin (clust) and C3b within the spinal cord (case MS55_SC). H1 shows vast immunolabelling of C1q within the peri-plaque. H2 and H3 show immunolabelling of cells morphologically resembling astrocytes within the plaque. H4 demonstrated C3b immunolabelling of neurones within the cortex. Scale bar is shown in G4 and is applicable for all plates. (TIFF 2 MB)

Additional file 5: Figure S4: Positive cellular and myelin immunolabelling in MS and controls. Percentage of sections with positive immunolabelling for each antibody in both cells and myelin is shown with error bars for multiple sclerosis (MS, 42 sections from 17 cases), non-neurological controls (C, 14 sections from 7 cases) and neurological controls (NC, 11 sections, from 9 cases). Significant results are shown by p value examining differences between the MS group and controls or neurological controls; differences between the control groups were not included for reasons of clarity. (JPEG 119 KB)

Additional file 6: Figure S5: Quantitative immunolabelling of cells from different plaque types. Quantification of immunolabelled cells is shown for each antibody comparing different plaque types; active (130 areas examined from 8 sections with active plaques), chronic active (180 areas examined from 9 sections with chronic active plaques) and chronic inactive (310 areas examined from 18 sections with inactive plaques). Groups show mean values +/_ standard error and significant results are shown by p values examining differences between active and chronic active groups, and chronic active and chronic inactive groups. (TIFF 1 MB)
