# Supplementary material for: Complement activation in multiple sclerosis plaques: an immunohistochemical analysis
Source: Acta Neuropathol Commun. 2014 May 9;2:53. doi: 10.1186/2051-5960-2-53 (PMC4048455; doi:10.1186/2051-5960-2-53)
Supplement: Supplementary file 1 — Additional file 1: Table S1: Antibodies used for immunohistochemistry. (PDF 95 KB) [file 40478_2014_129_MOESM1_ESM.pdf]

Supplemental table 1: Antibodies used for immunohistochemistry.

**Antibody name / no.**	**Antigen / Target**	**Type, species**	**Isotype**	**Source**	**Conc (µg/ml)**
C3/30	C3b, iC3b	MAb, mouse	IgG1	In-house	3
A209	iC3b	MAb, mouse	IgG2b	Quidel	6
HM2195	C3a receptor	MAb, mouse	IgG2b	Hycult Biotech	3
550733	C5a receptor	MAb, mouse	IgG1	BD Pharmingen	3
A252	Bb neo	MAb, mouse	IgG1	Quidel	3
A0136	C1q	PAb, rabbit	IgG	DAKO	4
A251	C4d neo	MAb, mouse	IgG1	Quidel	2
A213	C4d neo	MAb, mouse	IgG1	Quidel	60
B7	C9 neo (TCC)	MAb, mouse	IgG2a	In-house	30
MBI-6	Factor H-Tyr402	MAb, mouse	IgG1	In-house	19
MBI-7	Factor H-His402	MAb, mouse	IgG1	In-house	12
552886	Clusterin	MAb, mouse	IgG1	BD Pharmingen	2
C1inh	C1 inhibitor	PAb, rabbit	IgG	In-house	4.2
GFAP, M0761	Astrocytes, Ependymal cells	MAb, mouse	IgG1	DAKO	8
HLA-DR, M0775	Microglia	MAb, mouse	IgG1	DAKO	40
SMI 32, NE1023	NFL H non-phosphorylated	MAb, mouse, ascites	IgG1	Merck	1:1500
MOG Y10	Myelin	MAb, mouse	IgG1	In house	10
CD3, sc-20918	T cells	PAb, rabbit		Santa Cruz Biotechnology Inc	2
CD20, M0755	B cells	MAb, mouse	IgG2a	DAKO	1.8

Citraconic anhydride was used for antigen removal for all antibodies except B7, which required treatment with Proteinase K. MAb: Monoclonal antibody. PAb: Polyclonal antibody. Conc: concentration; shown in µg/ml except for SMI-32 a commercial antibody for which a dilution factor is shown. NFL: neurofilament. MOG: myelin oligodendrocyte glycoprotein.
