# Supplementary figures and images for: Complement activation in multiple sclerosis plaques: an immunohistochemical analysis
Source: Acta Neuropathol Commun. 2014 May 9;2:53. doi: 10.1186/2051-5960-2-53 (PMC4048455; doi:10.1186/2051-5960-2-53)

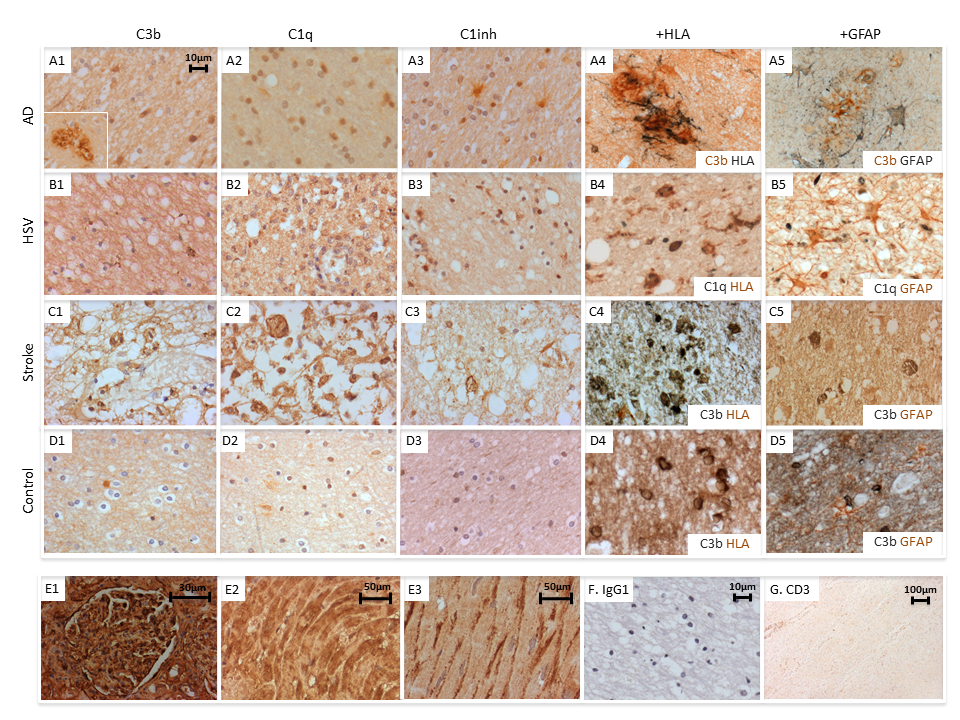

Supplement: Supplementary file 2 — Additional file 2: Figure S1: Control sections. Figures showing examples of complement immunolabelling in paraffin wax sections from white matter from cases of acute and chronic neurological inflammation and normal control white matter immunostained with antibodies to C3b, C1q and C1 inhibitor. Alzheimer’s disease (AD), cases A192 and A173 (plates A1-A5), shows minimal immunolabelling of complement except within the amyloid plaque (inset A1). Herpes Simplex Virus encephalitis (HSV), cases B5046-15 and B5125-9 (B1-5) shows more cellular and non-cellular immunolabelling of complement. Cellular immunolabelling of C3b (C1), C1q (C2) and C1inh (C3) is demonstrated in white matter of a case of ischaemic stroke (case 122891-b) with immunolabelling of macrophages and microglia within the infarcted tissue. Non-neurological control white matter, cases C041 and C038 (D1-5) showed minimal complement immunolabelling. Double labelling in both neurological and non-neurological control cases demonstrated colocalisation of C3b with HLA positive microglia (D4) but not GFAP positive astrocytes (D5). E1 demonstrates positive immunolabelling of C3b within the glomeruli of kidney sections with glomerulonephritis. E2 (C1q) and E3 (C1inh) demonstrate C1q deposition on the cardiac myocardial cells and C1 inh located on the sarcolemma in cardiac myocytes in section of myocardium from patients with myocarditis due to an adenovirus infection. Isotype anti-IgG1 (F, case MS64_B) and anti-IgG2 (not shown) were consistently immunonegative. MS tissue containing an active plaque (G) with a small diameter blood vessel demonstrated immunolabelling of T lymphocytes with in the blood vessel wall and occasionally within the perivascular space; minimal positive immunolabelling for anti -CD3 was is seen in the plaque. Scale bar is shown in A1 and is applicable for plate A, B, C and D. Scale bars for plates E, F and G are shown on each plate. (TIFF 2 MB) [file 40478_2014_129_MOESM2_ESM.tiff]

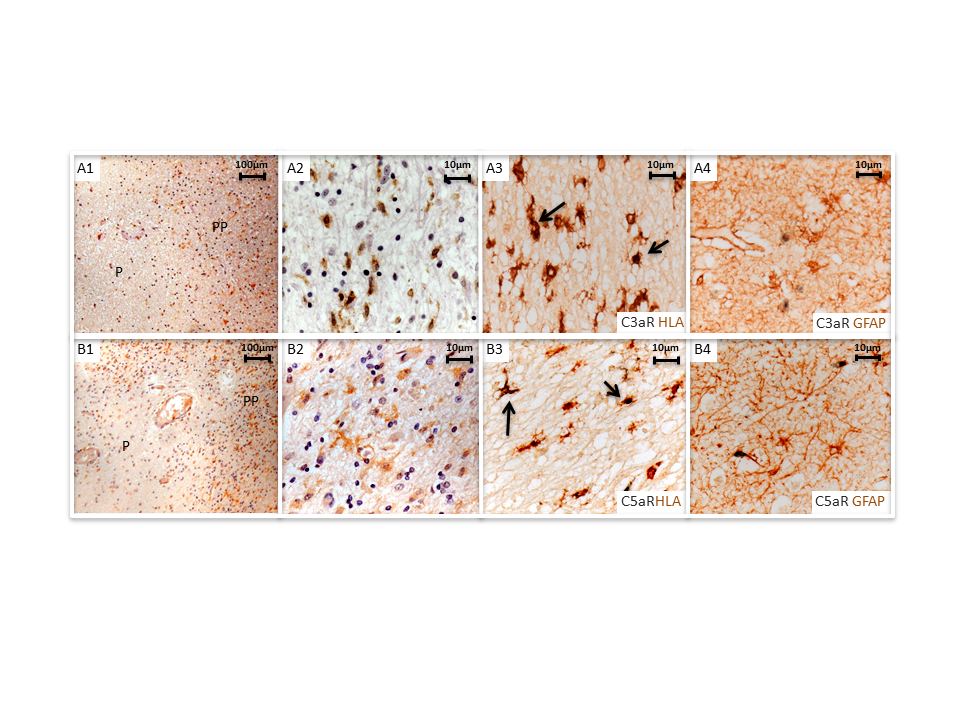

Supplement: Supplementary file 3 — Additional file 3: Figure S2: Complement anaphylatoxin receptors. Paraffin wax sections immunolabelled with anti-C3aR (A1-4) and anti- C5aR (B1-4). Figure A1 (case MS307_20, inactive plaque) and B1 (case MS230_S2, chronic active plaque) shows a low power image of the plaque (P) and peri-plaque (PP) with immunolabelled C3aR (A1) and C5aR (B1) cells. Immunopositive C3aR and C5aR labelled cells are seen predominantly at the lesion edge; C3aR demonstrated in A2 (case MS307_20, inactive plaque) and C5aR in B2 (case MS160_S3/1 chronic active plaque). Both C3aR (A3, case MS312_19, chronic active plaque) and C5aR (B3, case MS307_20, inactive plaque) (grey) were shown to co-localise with HLA-DR + microglia (brown, arrow). No co-localisation was shown with GFAP + astrocytes (brown); C3aR demonstrated in A4 (case MS312_19, chronic active plaque) and C5aR in B4 (case MS307_20, inactive plaque) (grey). Scale bars are shown for each slide. (TIFF 912 KB) [file 40478_2014_129_MOESM3_ESM.tiff]

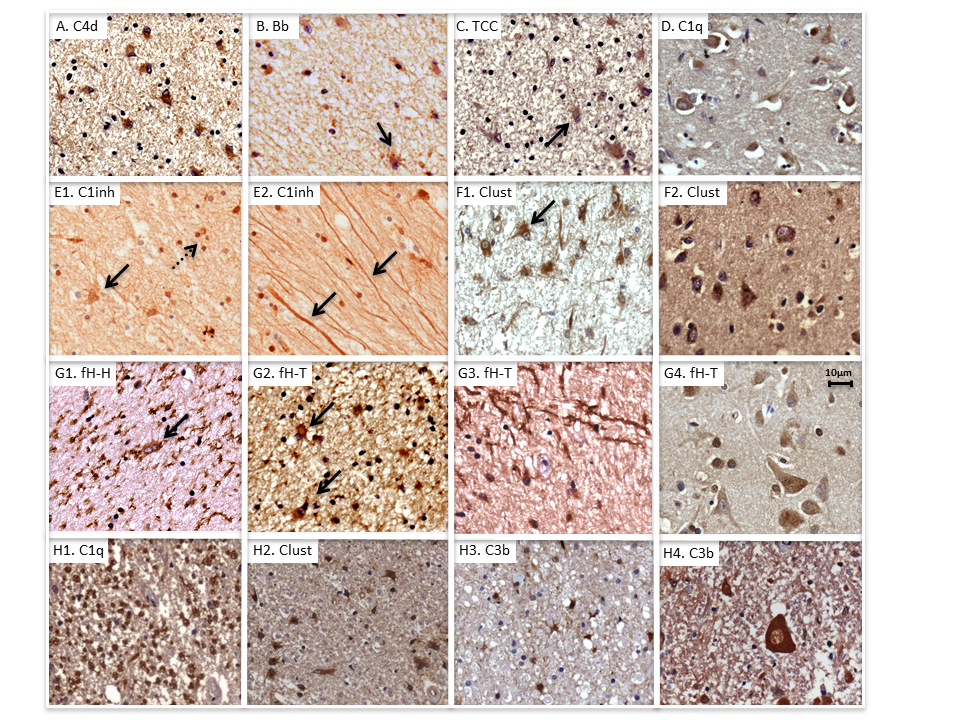

Supplement: Supplementary file 4 — Additional file 4: Figure S3: Complement antibody staining in MS. Plate A demonstrates paraffin wax sections showing anti-C4d immune-positive cells of astrocyte morphology in an active lesion (case MS179_B). Alternative pathway component Bb is shown in plate B on immunolabelled cells within a chronic active plaque (case MS225_S13; arrow indicates an immunolabelled cell of astrocyte morphology). C shows immunolabelling with anti-TCC demonstrating immune-positive cells with astrocyte morphology (arrow). D shows C1q immunopositive neurones in the cortex of case MS55_B. E1 and E2 show immunolabelling with anti-C1 inhibitor demonstrating immune-positive cells with astrocyte (E1, arrow) and microglial-like (E1, dashed arrow) morphology as well as immunolabelling on individual myelin sheaths (E2, arrow) (both case MS160_S1, active plaque). F1 and F2 (F1: case MS64, inactive plaque. F2: case MS128_B, cortex) show anti-clusterin immune-positive cells of astrocyte morphology (F1, arrow) and neurones (F2). Case MS179 is heterozygote for the factor H (fH) Tyr402His polymorphism. Immunolabelling with fh_His402 (fH-H) and fH-Tyr402 (fH-T) demonstrates immune-positive cells within the plaque (G2, fH-Tyr402, arrow) and peri-plaque (G1, fH-His402, arrow and G3, fh-Tyr402) with fragmented myelin shown in the peri-plaque areas. G4 demonstrated neuronal immunolabelling with fH-Tyr402 within the cortex (case MS64_B). Plate H1-H4 show immunolabelling of C1q, clusterin (clust) and C3b within the spinal cord (case MS55_SC). H1 shows vast immunolabelling of C1q within the peri-plaque. H2 and H3 show immunolabelling of cells morphologically resembling astrocytes within the plaque. H4 demonstrated C3b immunolabelling of neurones within the cortex. Scale bar is shown in G4 and is applicable for all plates. (TIFF 2 MB) [file 40478_2014_129_MOESM4_ESM.tiff]

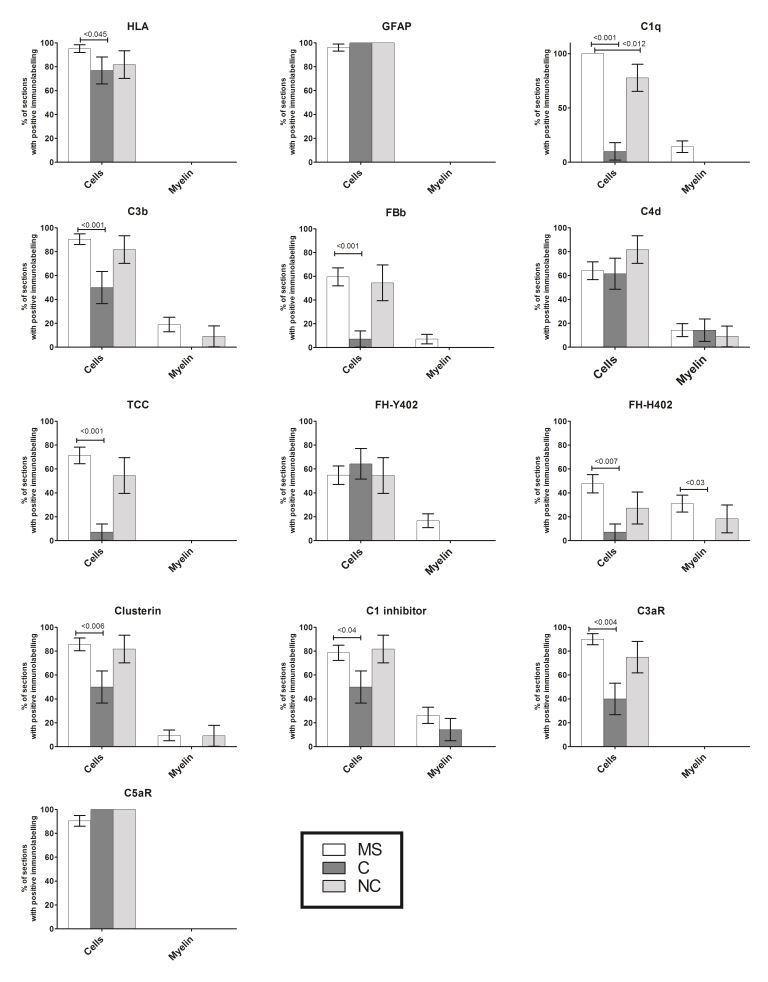

Supplement: Supplementary file 5 — Additional file 5: Figure S4: Positive cellular and myelin immunolabelling in MS and controls. Percentage of sections with positive immunolabelling for each antibody in both cells and myelin is shown with error bars for multiple sclerosis (MS, 42 sections from 17 cases), non-neurological controls (C, 14 sections from 7 cases) and neurological controls (NC, 11 sections, from 9 cases). Significant results are shown by p value examining differences between the MS group and controls or neurological controls; differences between the control groups were not included for reasons of clarity. (JPEG 119 KB) [file 40478_2014_129_MOESM5_ESM.jpeg]
